# Recent Advances in the Use of Green Corrosion Inhibitors to Prevent Chloride-Induced Corrosion in Reinforced Concrete

**DOI:** 10.3390/ma16237462

**Published:** 2023-11-30

**Authors:** Luca Casanova, Federica Ceriani, Elena Messinese, Luca Paterlini, Silvia Beretta, Fabio Maria Bolzoni, Andrea Brenna, Maria Vittoria Diamanti, Marco Ormellese, MariaPia Pedeferri

**Affiliations:** Department of Chemistry, Materials and Chemical Engineering “Giulio Natta”, Politecnico di Milano, Via Mancinelli 7, 20131 Milan, Italy; luca.casanova@polimi.it (L.C.); federica.ceriani@polimi.it (F.C.); elena.messinese@polimi.it (E.M.); luca.paterlini@polimi.it (L.P.); silvia.beretta@polimi.it (S.B.); fabio.bolzoni@polimi.it (F.M.B.); mariavittoria.diamanti@polimi.it (M.V.D.); marco.ormellese@polimi.it (M.O.); mariapia.pedeferri@polimi.it (M.P.)

**Keywords:** green inhibitors, rebars corrosion, plant extracts, waste materials, concrete, chloride-induced corrosion, bacteria

## Abstract

Inhibitors for the prevention of corrosion in reinforced concrete are chemical substances able to reduce carbon steel reinforcements corrosion without altering the overall properties of concrete. Today, many commercially available substances have a negative impact on human safety during either the inhibitor synthesis, their handling or application in field. Green corrosion inhibitors are nontoxic, biodegradable and environmentally biocompatible substances. They are generally made of extracts from natural plants or waste, which are abundantly available in several countries. The majority of green inhibitor molecules usually contain multiple bonds, aromatic rings, polar functional groups and electronegative atoms as P, N, S or O; the latter are able to coordinate with metal cations to form protective layers on the metallic surface of the reinforcements, so as to inhibit the development (initiation and/or propagation) of the corrosion process. In this review, the most recent achievements on the study and investigation of green corrosion inhibitors for concrete structures are presented and discussed. Inhibitors are classified based on their nature and inhibition mechanism. The inhibition effectiveness of the substances is compared with the well-established effective nitrite-based inhibitor, distinguishing between accelerated and long-term tests. Based on the available data, a summary of corrosion inhibitors efficiency is reported.

## 1. Introduction

Concrete is one of the most ancient composite materials ever used by humankind for structural purposes. It is generally constituted by a mixture of cement, inert aggregates—like gravels and sand—and water. Cement is mainly composed of agglomerates of tricalcium silicate (C_3_S), dicalcium silicate (C_2_S), tricalcium aluminate (C_3_A) and tetracalcium aluminoferrite (C_4_AF). Those constituents are responsible for imparting, upon complex hydration reactions, the development of mechanical resistance—thanks to the formation of the calcium silicate hydrate (C-S-H) gel—and the rise of alkalinity (pH ~13) [1,2]. This environment is particularly beneficial in the case of reinforced concrete, where carbon steel rebars are generally inserted in order to impart resistance against tensile loadings. In fact, in high-pH environments, carbon steel spontaneously covers with a passive layer constituted of hematite (α-Fe_2_O_3_) and magnetite (Fe_3_O_4_) protecting the metal from the contact with aggressive species responsible for both generalized attacks and more dangerous localized attacks. Most of the rebars localized corrosion is related to the presence of de-icing salts, frequently applied in winter, or contact with seawater in structures like bridges and highways, where the risk of localized corrosion with severe corrosion rate becomes very high, involving often-tragic consequences.

The risk of corrosion is even amplified if the alkalinity inside concrete is compromised: in fact, at a neutral pH carbon steel loses passivity and corrodes uniformly under the action of oxygen and moisture. Among the possible phenomena responsible for this occurrence, the detrimental action imparted by CO_2_ and SO_2_ is particularly critical. Indeed, when carbon dioxide diffuses inside concrete and reacts with hydration products, it converts alkaline species like Ca(OH)_2_, and to a minor extent even C-S-H gel [3] and calcium aluminates, into CaCO_3_. Similarly, SO_2_ reacts with calcium-bearing compounds, resulting in their sulfonation [4]. Both carbonation and sulfonation may decrease the pH below the threshold of iron oxide stability, compromising their protective efficiency and leading either to generalized corrosion or increasing the probability of chloride-induced corrosion and eventually compromising the mechanical stability of the structure. 

As mentioned above, apart from uniform corrosion occurring in carbonated concrete upon exposure to oxygen and water, the worst enemy of metallic rebars is chloride-induced corrosion, given its generally fast propagation, short initiation time and the difficulties detecting it by visual inspection. Up to now, the vast majority of the models assume corrosion to occur on carbon steel once the concrete pore solution exceeds a chloride content around 0.4 wt.% with respect to Portland cement weight in aerated concrete [1,5,6]. 

The most diffused interpretation of the corrosion kinetics in concrete is the model developed by Tuutti [7], where incubation and propagation times are deliberately separated as both processes present different kinetics and rate-determining factors. Considering chloride-induced corrosion, the model states that once in a local area the critical chloride content is reached, an anode readily forms due to the local dissolution of the passive film, causing the formation of a macrocell. Here, local voltages, up to several hundreds of millivolts, may be established between the negative depassivated anode and the positive cathode, even if several centimeters apart. The corrosion rate depends on the availability of oxygen at the cathode and on the presence of moisture, and consequent concrete pore solution resistivity. As a result, there is the onset of very high corrosion rates, in the order of several millimeters per year, which may compromise the structural integrity of the metal. 

Nowadays, several strategies are available on the market for reducing or even eliminating the danger of corrosion attacks, considering both passive and active techniques. Cathodic protection and prevention [8] belong to the latter method: rebars are polarized cathodically and several beneficial effects occur, like (1) a negative potential shift, (2) the development of alkalinity over the electrode surface and (3) electrophoretic migration of corrosive anions far from the rebars, ensuring a higher longevity of the structure. Alternatively, the selection of corrosion-resistant rebars, the use of fiber-reinforced polymer (FRP) bars, the application of surface coatings or the use of corrosion inhibitors [9,10,11,12,13,14] are well-recognized strategies to mitigate reinforced-concrete degradation. Thanks to the corrosion-inhibitive properties of many substances, it is possible to delay the incubation time and also to reduce the corrosion rate of many forms of corrosion.

Depending on the interaction mechanisms, with the ongoing corrosion reaction, the inhibitor may control the anodic kinetics, suppressing metal dissolution, the cathodic reaction, limiting the amount of cathodic reactant interacting with the metal, or both. The latter effect may be evaluated by performing a linear polarization experiment (Tafel analysis ± 250 mV/corrosion potential) to verify electrochemical parameters like corrosion potential (E_corr_), anodic Tafel slope (b_a_) and cathodic Tafel slopes (b_c_). Anodic inhibitors are responsible for enhancing E_corr_ nobility and increasing b_a_ beyond common values encountered during iron dissolution; on the other hand, cathodic retardants decrease E_corr_ and increase b_c_. Generally speaking, organic inhibitors work as a mixed inhibitor [7]. Mixed-type inhibitors are intended to be those molecules capable of providing an inhibitive effect towards both the anodic and cathodic reaction kinetics. The adsorbed molecules, in fact, may provide a barrier to the anodic dissolution of the metal and to oxygen reduction reaction by limiting the availability of oxygen at the adsorption sites. 

Following this route, nitrites are nowadays recognized as the benchmark for the corrosion inhibition of metallic rebars placed in concrete owing to their high efficiency and economic affordability. The mechanism behind the inhibitive property of nitrites is of the anodic type, as it forms a passivation film at the metal surface, and can be summarized according to Equations (1) and (2) [7,15,16]:

(1) $$2{Fe}^{2+}+2OH^{-}+2NO_{2}^{-}\to 2NO+{Fe}_{2}O_{3}+H_{2}O$$

(2) $${Fe}^{2+}+{OH}^{-}+NO_{2}^{-}\to NO+\gamma FeOOH$$

Nitrites are highly competitive with chlorides in reacting with weak spots and defects present in the iron oxide layer, promoting an efficient healing process. Since the oxide layer is generally a few nanometers thick, nitrite consumption is generally relatively low. 

Despite those advantages, important safety and environmental concerns require new substances to be developed as valuable alternatives to nitrite-bearing compounds. Nitrites, in fact, are well known pollutants for both the human and aquatic environment as they promote the growth of algae, which are toxic for fishes and humans. A relatively recent class of environmentally sustainable molecules, generally referred to as “green inhibitors”, is gaining attention among both the industrial and the academic communities, along with several other strategies to reduce the carbon footprint of reinforced concrete, as the addition of natural fibers and organic waste compounds in the mix design phase [17]. Green inhibitors are natural source-based substances generally considered eco-friendly, nontoxic, and biodegradable [18]. Generally, the two mechanisms involved during metal corrosion inhibition are the transport of the green inhibitor’s molecule to the rebar and its binding with specific metal adsorption sites. Green inhibitors may interact with the rebar surface chemically or physically depending on their specific structure. The inhibitor’s molecules mostly contain heteroatoms, aromatic rings, and unsaturated bonds and multiple bonds with p-electrons, which are prone to be transferred to the free *d*-orbitals of iron, leading to chemisorption due to a donor–acceptor interaction. Heteroatoms could also be involved in physisorption, together with polar functional groups, which may electrostatically interact with the surface of steel reinforcements, maybe placing hydrophobic chains of the molecules perpendicularly to the metal. This may help prevent oxygen and water from reaching the carbon steel surface, thanks to the formation of a monolayer of molecules or to the stabilization of the passive film. Since the addition of a corrosion inhibitor may modify the composition of the electrical double layer, common electrochemical techniques like electrochemical impedance spectroscopy (EIS) and linear polarization resistance (LPR) may be adopted to estimate electrode capacitance and so to monitor the adsorption efficiency of the molecule. 

Green inhibitors produced from organic and waste stockpiles are currently under investigation both as admixtures and as paints. Following this idea, extensive studies already exist on the effect of Mussel Adhesive Protein (MAP) [19,20,21], as well as several plants extracts [22,23], employed as paint on reinforced concrete rebars, investigating the interesting self-healing effects of the protective films formed by these organic proteins. Silica fume, a common by-product of several industrial processes, has been investigated in similar studies [24]. However, this specific green corrosion inhibitors will not be further explored in this review, since the protection mechanism substantially differs from the mix design additives.

The aim of this review is to critically analyze the effectiveness of some organic substances referred to as green inhibitors and compare their efficacy in contrasting the chloride-induced corrosion of reinforced concrete with that of nitrite-based molecules. Only recent papers (last 10 years) are selected, depending on the availability of the following information: (1) the mix design of the mortar/concrete, (2) the composition and pH of the synthetic concrete pore solution, (3) the duration of the experiment, (4) the inhibitor concentration, (5) the chloride content (preferably with respect to cement weight), (6) the critical chloride content in presence of inhibitor, and (7) the inhibition efficiency (IE%). The latter parameter, in the surveyed literature, is generally evaluated electrochemically as (1) a reduction in corrosion current density (*i_corr_*), or (2) an increase in polarization resistance. All these quantities basically reflect a measure of the decrease in the corrosion propagation rate; unfortunately, this makes the analysis of the inhibitor efficacy incomplete, as in chloride-induced corrosion an increase in initiation time would be even more valuable. Eventually, a final discussion comparing green molecules with commercially available concrete inhibitors is provided. 

## 2. Botanical Green Inhibitors

### 2.1. Plant and Leaf Extracts

Much attention today is paid to the synthesis of inhibitors available from natural resources. With this regard, plant extracts constitute the vast majority of green inhibitors. Plant extracts contain so-called phytochemical components (as alkaloids, flavonoids, tannins, steroids, glycosides, phenols, carbohydrates, amino acids, proteins, ascorbic acid, phytosterols, monosaccharides…), which are active principles generally containing heteroatoms, lone pairs, aromatic rings, π-electrons, and active groups, such as –OH, –COOH, and—NH_2_ [25,26]. Then, phytochemicals are able to interact with metallic ions, leading to the formation of a protective layer on the metal surface that hinders the anodic and/or cathodic reactions, inhibiting corrosion. The interaction between phytochemicals and the metal surface can be physical or chemical. In the first case, the inhibitory effect is lower, since physisorption is generally weak. On the contrary, chemisorption is a strong type of interaction that could occur, for example through chelation and hydrogen bonding, leading to an enhanced protective effect [26,27].

Extracts can be obtained by different methods from several parts of plants and trees in particular leaves, since they are the sites of photosynthesis responsible for phytochemicals production [26,28].

The extraction process significantly influences the final properties and quantity of the obtained phytochemicals. In particular, the selection of the proper solvent plays a key role in the extraction process. Solvents are chosen according to their polarity, which should be the same as the phytochemical compound to be extracted. The most common solvents comprise water, methanol (MeOH), ethyl acetate (EtOAc), dichloromethane (DCH), hexane (hex), and acetone [27,29].

#### 2.1.1. Tests in Solution

The influence of the solvent selected for the extraction process was studied in the case of *Olea europaea leaves* extract, testing the use of (hex), (DCH), (EtOAc), and (MeOH), which displayed an increasing polarization index [30]. IE% was evaluated starting from *i_corr_* measured by Tafel analysis performed in a NaOH (0.1 M) + NaCl (0.5 M) solution. The higher the polarity of the employed solvent, the higher the IE%, reaching the maximum value of 91.9% with methanol. This is probably due to the fact that phytochemicals contained in olive leaves mainly consist of phenols and flavonoids, which are polar compounds; thus, with methanol, the extraction process was promoted.

Another example of using different solvents is associated with corrosion inhibitors extracted from *Rosa damascena* leaves and flowers using ethanol (RDEE) and sulfuric acid (RDAE), and water as solvent [26,31]. Concerning the leaf extract, it is observed by Anitha et al. [26] that, when added to a simulated concrete pore solution (SCP) containing Cl^−^ (0.5 M NaCl), it efficiently protects the rebars forming an adsorbed layer, following a Langmuir isotherm, which limits Cl^−^ diffusion. The IE% evaluated by EIS and Tafel tests reveals some differences in the protective effect provided by RDEE and RDAE, the latter showing a slightly higher anticorrosive effect. Both inhibitory compounds cause an increase in charge transfer resistance, R_t_, which becomes larger for higher inhibitor concentrations on account of better surface coverage. The same trend appears from Tafel analysis, with *i_corr_* decreasing for larger inhibitor concentration. 

The influence of inhibitor concentration on the anticorrosive effect of plant extracts is evaluated also in [32] with the use of *Davidia involucrata* leaves extract (DILE). Five different concentrations (0, 10, 50, and 100 mg·L^−1^ of DILE) are added to a simulated pore solution containing 3.5 wt.% CaCl_2_. Again, the larger the concentration of leaves extract, the higher the corrosion inhibition effect. At the greatest content of DILE (100 mg·L^−1^) *i_corr_* decreases from 62 μA·cm^−2^ measured in the reference solution to 3 μA·cm^−2^, corresponding to an IE% of 96%. This study highlights that another parameter influencing the IE% of DILE is the temperature. In fact, by increasing the temperature from 25 to 35 °C the IE% increases by about 5% due to a promoted chemisorption of phytochemicals on the carbon steel surface, which occurs mainly in anodic sites of carbon steel surfaces to reduce iron dissolution: the action is therefore mainly exerted on the anodic semi reaction. However, Tafel results show small changes also of b_c_; thus, DILE can be assumed to be a mixed-type inhibitor.

*Prosopis juliflora* extract is also proposed as mixed inhibitor [33]. The leaf, prior to test, is washed dried and then immersed with methanol for 6 h and the solution is left overnight in order to allow the extraction of the active principle. The active principles present in such extract act on both anodic and cathodic semi reaction following two main mechanisms. The first one, as already mentioned, involves heteroatoms and aromatic ring π-electrons, which are adsorbed on the carbon steel surface, hindering the anodic process. 

The second mechanism implied is a consequence of Cl^−^ adsorption on the metal surface that consequently acquires a negative charge. At this point, the protonated molecules present in the leaf extract are adsorbed on carbon steel due to an electrostatic effect partially hindering the availability of cathodic reactant at the metal surface. 

*Fatsia japonica* leaf extract (FJLE), instead, is an anodic-type inhibitor, as clearly demonstrated by the increase in b_a_ reported by Wang et al. [34], when FJLE is added to SCP containing 35 g·L^−1^ of Cl^−^. Starting from Tafel results, an estimation of IE% is obtained, showing once again that the highest protection efficiency (88.1%) occurs using the largest concentration of inhibitor (1000 mg·L^−1^). The efficiency of the inhibitory effect is also evaluated at different immersion times, from 24 to 168 h, and a progressive increase in IE% is observed. This trend corresponds to a continuous increase in R_t_, evaluated through EIS analyses, and demonstrates the long-term protective ability of FJLE. EIS results also highlight a highly capacitive response of the adsorbed FJLE film on the carbon steel surface, as demonstrated by the slope of the impedance modulus |Z| in the Bode diagram, which is around 1 in the frequencies middle range.

**Figure 1:**
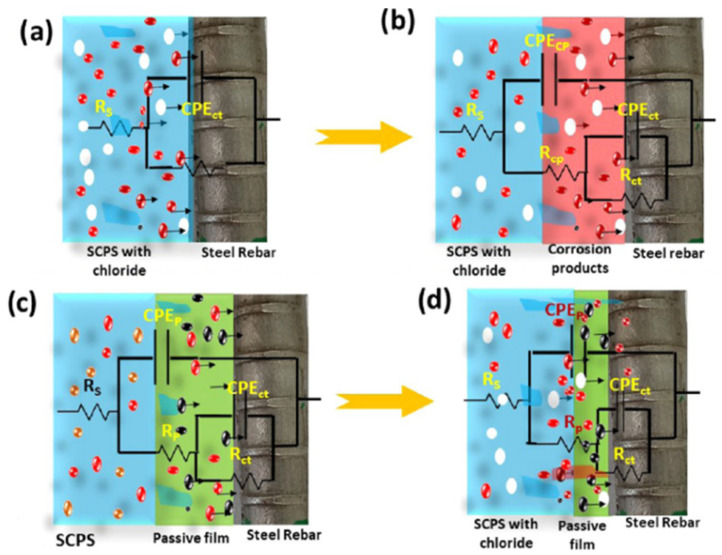
Electrochemical equivalent circuits used to fit EIS spectra after (**a**,**b**) exposure to Cl^−^ without and with inhibitor (**c**,**d**) (reprinted with permission from [35]).

A similar inhibitor concentration is required for an inhibitor extracted from *Conifer cone* (Pinus resinosa) [35], tested in SCP with the addition of 30 g·L^−1^ of NaCl. A dosage of 1 g·L^−1^ permits a raise of the IE% to 81.2%, evaluated by EIS, according to the equivalent circuits proposed in Figure 1. 

This is possible thanks to moieties like −C=O, −C−N, −OH, −CHn, C=C and aromatic rings, which are prone to donate unpaired electrons to empty Fe *d*-orbitals.

Liu et al. [36] revealed the inhibitive properties of a ginger extract, considering the corrosion of carbon steel immersed in a saturated Ca(OH)_2_ solution (pH 12.5). However, its inhibitive properties are evaluated only qualitatively, attributing the inhibitive effect to adsorption onto the metal of carboxylic, hydroxyl and aliphatic groups, finding an optimum considering a dosage of 2 wt.% in presence of 0.1 M NaCl.

Mango extracts may also serve as effective corrosion inhibitors. Rahmani et al. [37] undertook a detailed computational and experimental study clarifying its mechanism of inhibition. In particular, it was found that positively charged mango extracts may (1) migrate in correspondence of the pitted region, resulting in physical adsorption, (2) form large agglomerates of molecules thanks to the bridging action of Ca^2+^ and K^+^ ions present in solution and (3) form chemical interactions with the substrate according to the donation of lone pairs localized on heteroatoms and π-electrons. With a dosage of 2 wt.% in a solution containing 3.5 wt.% NaCl, the authors declared an IE% of 98%. 

*Phytates* are phosphorous-containing molecules naturally present in many hulls and kernels of seeds, already used for the corrosion inhibition of Cu [38] and Mg [39] alloys. Liu et al. [40] tested the inhibitive properties of 0.01 M sodium phytate on carbon steel immersed in SCP with 0.3 M NaCl. The presence of phosphate and hydroxyl functional groups allows an overall IE% of 68.3%; phytates preferentially adsorb on carbon steel and chelate with Fe^2+^ centers, preventing contact with Cl^−^.

**Figure 2:**
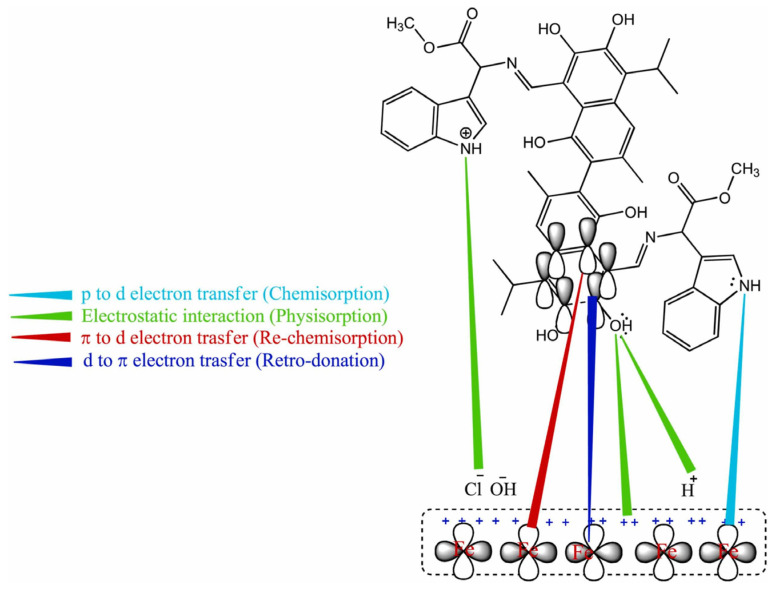
Pictorial representation of the GIM inhibition mechanism on low-carbon steel in 1 M NaOH + 1 M NaCl solution (reprinted with permission from [41]).

The Gossypol–indole modification (GIM) corrosion inhibitor was tested for low-carbon steel in a 1 M NaOH + 1 M NaCl solution [41]. GIM molecules have a planar structure, which promotes the interaction of the active sites (hydroxyl, amino, methoxy and benzoyl rings) with metal ions. Thus, active complexes were formed between the GIM inhibitor and iron ions; moreover, electron pairs could be shared from the polarized GIM molecules to the vacant *d*-orbitals of iron. As a result, a protective film was produced on the carbon steel surface, as shown in Figure 2. The presence of such an adsorbed layer led to a significant reduction in *i_corr_*, from 70.95 mA·cm^−2^, measured in the reference solution by potentiodynamic polarization, to 1.36 mA·cm^−2^ at a GIM concentration of 100 mg·L^−1^. The inhibitory effect increases for larger GIM concentrations, while it becomes lower at increasing temperatures, since GIM molecules’ interaction with the metal surface is not only chemical but also physical via electrostatic interactions, which are affected by high temperatures. 

As a note on this work, despite *i_corr_* being decreased by more than one order of magnitude, this value cannot be considered acceptable, as 1.36 mA·cm^−2^ roughly corresponds, in carbon steel, to a corrosion rate of 16 mm·year^−1^. 

A particular type of plant from which green inhibitors could be extracted is represented by succulent plants. *Opuntia ficus-indica* extract has been studied as a corrosion inhibitor in SCP containing different NaCl concentrations (0.5, 1, 2, 4, 8 and 16 g·L^−1^). This cactus mucilage is a carbohydrate containing arabinose, galactose, galacturonic acid, rhamnose and xylose residues. The −OH groups present in the polysaccharide are deprotonated in the alkaline environment and become active sites for the interaction with iron ions on the metal surface, forming an insoluble film on it, whose thickness increases with the extract concentration. The generation of such a protective layer is associated with an IE% above 90% for a NaCl concentration from 4 to 16 g·L^−1^ [42].

#### 2.1.2. Tests in Concrete

Several studies have been performed directly in concrete samples rather than in a simulated pore solution.

An example is provided by one investigating *Bambusa Arundinacea* leaf extract, which was added to concrete samples (mix design reported in Table 1) containing 0.94 wt.% of Cl^−^ by cement mass [43]. The inhibition mechanism was quite different with respect to the previous ones. In fact, it was reported that corrosion inhibition was derived from the hydrophobic effect of the extract. The fatty carboxylate present in the green inhibitors could combine with Ca(OH)_2_, producing hydrophobic salts that clog the pores of the carbon steel passive layer, preventing the formation of differential aeration cells and the diffusion of oxygen.

When using *Ricinus communis* [44] leaf extract, the same type of dual mechanism observed in [33] is assumed to occur. In particular, the hydrated active compound of the green inhibitor attracts the excess Cl^−^, binding to them and forming a barrier layer adsorbed on the carbon steel surface that hinders both anodic and cathodic reactions. The best efficiency was provided by the largest inhibitor concentration (100 ppm), and it increased with time. According to EIS, R_t_ grows by about 200 Ω·cm^−2^ from 30 to 120 days of test, corresponding to an IE% enhancement from 47 to 87%.

Matter et al. [45] proposed the inhibitive properties of long-chain carboxylic acids extracted from vegetable oils of *coconut* and *sunflower* fatty acids. These inhibitors are tested both in solution (SCP, pH 12.5–13 and 1 M NaCl) and in concrete considering a cement:sand:water ratio of 1:3:0.50 and a chloride content of 3 wt.%. All the molecules are found to interact with the substrate through −NH_3_ and −COOH groups, imparting IE% between 85 and 96% with a dosage of 0.1 g·L^−1^.

Naderi et al. [46] evaluated the effect of *Glycyrrhiza glabra*, an extract of *Licorice*. Even if the molecule alone demonstrated a slightly lower IE% compared to nitrites, a synergistic effect was verified when mixing both inhibitors (50:50), with an IE% between 55 and 60% after 90 days in a mortar produced with cement IV, with a water:cement:sand ratio 0.5:1:3, containing 0.8 wt.% of NaCl vs. cement weight. The same authors investigated the inhibitive effect of a nettle extract (*Urtica Dioica*) [47], which was found to contain many organic molecules possessing S, O and N heteroatoms. Considering the most effective dosage (0.075 wt.%), an IE% of 77% was obtained in SCP containing 1 wt.% NaCl. The extract was demonstrated to inhibit the anodic reaction by precipitating in correspondence of pitted regions. 

Argiz et al. [48] considered the addition of ascorbic acid in different amounts, finding an optimum at 0.009% vs. cement weight for a chloride content equal to 3.29% vs. cement weight. Ascorbic acid is a derivative of glucose naturally present in many citrus fruits. The observed IE% was very similar to the one offered by nitrites, demonstrating ascorbic acid as a valid strategy for rebars protection. The molecule was assumed to interact with Fe^3+^ centers, present in γ-FeOOH, forming strong complexes promoting the formation of a barrier for further ferrous ion diffusion towards the electrolyte. 

Zhang et al. [49] performed an interesting study on rebar inhibition using *maize gluten meal* in mortar (water, cement, and sand in a ratio 0.6:1:3), introducing 3% NaCl vs. cement weight. An inhibitor dosage of 3% vs. cement weight was found to increase the IE% beyond 95% for a total test duration of 17 months, a result very competitive with commercial nitrites and amines. However, the concrete mechanical performances should be tested considering the high inhibitor quantity used during the experimental procedure, which can compromise the structural response of the material. 

**Figure 3:**
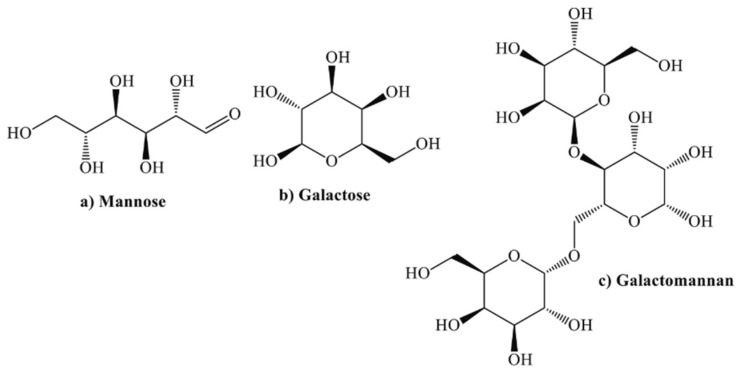
Guar gum extract chemical composition (reprinted with permission from [51]).

Thanks to the presence of many −COOH functional groups, as reported in Figure 3, even *guar gum* extracts [50] have been found to be effective inhibitory molecules for the protection of rebars in mortars. In particular, 1.4% of guar gum extract vs. cement weight can impart an IE% of 71.1%, in the presence of 3.5% NaCl vs. cement weight, thanks to the inhibition of the anodic reaction kinetics.

*Azadirachta indica* (neem) leaf extract [51] is used for corrosion inhibition tests performed in concrete samples exposed to a 3.5 wt.% NaCl solution for 128 days. In contrast to other studies, the authors investigate the inhibitive effect of the molecule in delaying chloride ion penetration. The protective effect of this inhibitor is related to both the formation of a physical barrier (physisorbed film), which prevents the contact of the carbon steel surface with Cl^−^, and to a chemical interaction with the metal that inhibits the anodic reaction. Indeed, *Azidarachtine* molecules contain several O heteroatoms, whose lone pair could interact with Fe^2+^ ions, resulting from rebars corrosion forming a chemisorbed protective layer.

Hadi et al. [52] studied the use of reed (scientific name *Phragmites Australis*) leaves extract (RLE). This green inhibitor mainly contains lignans, alkaloids, flavonoids, and O-substituted aromatic amines. The IE% evaluation was performed through Tafel tests carried out in mortar samples immersed for 180 days in a 3.5 wt.% NaCl solution. It was found that for increasing inhibitor concentration, the efficiency grows up to 76.98% at 0.5% RLE vs. cement weight. 

Even antioxidants from green tea were found to have inhibitory effect for corrosion of carbon steel in chloride-contaminated concrete. This anticorrosion effect is attributed to catechin derivatives, which represent the most abundant type of phytochemicals in green tea. The effect of such antioxidants is tested for mortar specimens exposed to wet-dry cycles in a 3.5 wt.% NaCl solution. Green tea extract acts as a mixed corrosion inhibitor, increasing the polarization resistance and reducing the corrosion rate of carbon steel, showing an efficiency ranging between 75 and 80% depending on the specific concentration of the inhibitor (from 20 to 40 L·m^−3^) [53]. 

*Cymbopogon citratus* leaf extract inhibition efficiency is verified by Okeniyi et al. [54] on NaCl-immersed concrete reinforced with carbon steel rebars. It is found that a leaf extract concentration of 0.083% vs. cement weight is sufficient to guarantee an IE% equal to 99.35%. The inhibitory effect of *C. citratus* is compared with that of NaNO_2_, revealing for equal mass of inhibitors that the leaf extract admixture generally offers a higher IE% than the NaNO_2_ admixture in the chloride-contaminated concrete.

The same research group has investigated the use of *Phyllanthus muellerianus* leaf-extract as corrosion inhibitor for carbon steel reinforcement corrosion in concrete immersed in 3.5 wt.% NaCl solution [55]. Physisorption of the organic molecules present in such an extract occurred according to Langmuir adsorption isotherm thanks to the presence of lone–pair and/or π-electrons on the aromatic rings, N, S, and Br heteroatoms and O contained in the organic compounds. After testing various concentrations, it was found that by adding 0.33% vs. cement weight of *Phyllanthus muellerianus* extract, an IE% = 97.58% is obtained.

Chemisorption is instead observed when studying *Anthocleista djalonensis* leaf extract [56]. The aromatic compounds present in the extract shared π-electrons with the vacant *d*-orbital of iron. However, even in this case, physical adsorption could occur due to electrostatic interaction involving the N-atoms of the amines and the C=O of the carbonyl compounds of the organic molecules and the charged carbon steel surface. The largest reduction in corrosion rate and the highest IE% (97%,) are obtained for a green inhibitor concentration of 0.417% vs. cement weight; decreasing the extract content to 0.167% the efficiency decreases to IE% = 81%; then, the inhibitory effect of the *Anthocleista djalonensis* extract remains good even at a low concentration.

The corrosion inhibition effect of extracts obtained from *Morinda citrifolia* (Noni) leaves extract was studied on reinforced concrete samples immersed in a 3 wt.% NaCl solution for 90 days. The inhibitory effect was mainly attributed to the –OH groups in the aromatic rings of flavonoids and ascorbic acid, which interacted both physically and chemically with the metal surface, forming a protective layer and increasing the Cl^−^ penetration resistance. An inhibition effect was found for an extract content larger than 0.17% vs. cement weight, for which IE% = 58%. In spite of a good IE%, the corrosion rate was quite high, presenting an optimized value around 0.1 mm·year^−1^, making the inhibitor unsuitable for practical applications. Such a high value may be related to the aggressiveness of the seawater-simulating solution used for the corrosion testing of carbon steel and to the mild effectiveness of the inhibitor used [28]. 

Green corrosion inhibitors could be extracted even from bark of plants, as in the case of bark extract admixture from *Rhizophora mangle* L. extracted using methanol [57]. Different concentrations of the extract were tested as corrosion inhibitors for carbon steel rebars embedded in concrete immersed in a 3.5 wt.% solution of NaCl. The maximum efficiency of about 99% was obtained for an inhibitor concentration of 0.467% vs. cement weight. In this case, the reduction in corrosion rate is due not only to the inhibitory capability of the phytochemicals present in the extract, but also to the presence of ester groups that hydrolyze in the alkaline environment, forming hydrophobic salts that hinder water penetration into concrete. 

*Opuntia ficus-indica* extract was also tested in concrete. The addition of Nopal slime in Cl^−^-contaminated mortar samples exposed to wet–dry cycles resulted in R_p_ values (measured by linear polarization resistance tests) one order of magnitude higher with respect to those obtained without the green inhibitor, corresponding to a maximum efficiency of about 80% [58].

### 2.2. Green Inhibitors from Agricultural Wastes

Among the most appealing opportunities to obtain green corrosion inhibitors with a low carbon footprint, the possibility of exploiting agricultural wastes stands out. Many materials that are commonly considered waste in our society are indeed potentially rich in functional groups (such as aldehydes, ketones, amines, polyamides, flavonoids, alcohols, aromatics, phenols…) which can make those molecules protective for carbon steel reinforcements in concrete. These inhibitors, thanks to their origin, are mostly harmless to the surrounding environment, allowing a widespread employment without incurring in serious safety restrictions.

**Figure 4:**
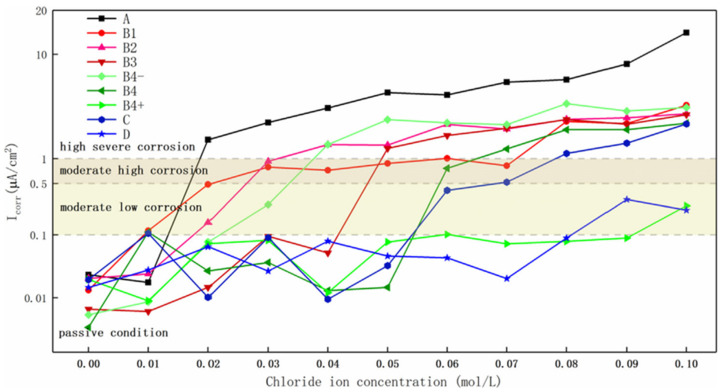
*i_corr_* as a function of chloride ion concentration calculated through linear polarization. A SCP solution blank system; B1 3 wt.% extract 1#; B2 3 wt.% extract 2#; B3 3 wt.% extract 3#; B4−, B4, B4+, respectively 1, 3 and 5 wt.% extract 4#; C 3 wt.% DMEA; D 1 wt.% sodium nitrite (reprinted with permission from [60]).

Song et al. [59] recently proposed the possibility of recycling the deciduous leaves of the *Platanus acerifolia*, widely planted in the urban landscapes worldwide, which would be otherwise treated as municipal waste, disposed through landfill and incineration. The *Platanus acerifolia* is indeed known for its abundant content of flavonoids, such as kaempferol and luteolin, which possess highly delocalized electrons, oxygen heteroatoms and suitable spatial configuration for metal cation chelation, embodying great potentials for application in the anticorrosion field [60]. For these reasons, *Platanus acerifolia* leaf (PAL) extract is deemed a potential mixed-type corrosion inhibitor on concrete rebars. Various extraction methods are employed on PAL, and the most efficient one is the ultrasonic-assisted alkali-pretreated ethyl extraction method. The extract was tested in saturated limewater at pH 12.5 with a dosage of 5 *v*/*v*%, improving the chloride threshold four-fold, considering the corrosion onset in the range of *i_corr_* = 0.1~0.5 µA/cm^2^ (Figure 4). 

The inhibiting effect of various PAL extracts is compared with more commonly employed corrosion inhibitors as sodium nitrite and dimethylethanolamine (DMEA, mixed type inhibitor), which are already widely available on the market and used as a benchmark. The protection effect of 5% PAL extracted with ethyl solution (Figure 4 B4+) measured through linear polarization is equivalent to sodium nitrite in the whole range from 0.01~0.1 mol·L^−1^ of chlorides, which is a promising result. Also, corrosion inhibition efficiency is measured through EIS up to values of 99% in the presence of 0.1 mol·L^−1^ of chlorides. 

*Conifer cones* (CCs) of the *Pinus resinosa* plant, renowned for their antioxidant properties since ancient time in the pharmaceutical field, contain α-pinene, β-pinene, D-limonene, myrcene, and β-caryophyllene, which are all functional groups with alleged corrosion inhibition properties. However, their use is limited and are mostly considered waste material. For these reasons, *Pinus resinosa* CCs were studied by Subbiah et al. [35] as stockpile for green inhibitor extraction through ethanol. The powders obtained after the extraction process were added in a concentration of 1 g·L^−1^ to a chloride-contaminated SCP and displayed an outstanding inhibitory efficiency of 80.7% after 720 h of immersion, compared to the reference samples.

Following the same principle, other agricultural and food wastes rich in potential inhibitors can also be considered as green supplies, such as orange peels and rice husks; still, studies on these substances appear to be still unripe and in strong need of further scientific validation.

## 3. Microbial Green Inhibitors

In recent years, a vast range of green inhibitors has been explored in order to define a novel approach to mitigate chloride-induced corrosion in reinforced concrete. In particular, the so-called microbial inhibitors have been analyzed, with a particular focus on those derived from bacteria. Microbial inhibitors involve the use of different microorganisms to repair microcracks, which serve as essential conduits for several aggressive substances, like chlorides. Bacterial corrosion inhibitors exploit the metabolic activities of bacteria to proactively repair and shield concrete, thus blending structural engineering and microbiology. Notably, the Microbial-Induced Calcite Precipitation (MICP) process has garnered substantial attention as a predominant method in this innovative field. Engaging the metabolic processes of specific strains of bacteria, MICP triggers the precipitation of calcium carbonate (CaCO_3_) within the concrete matrix. This biological activity not only repairs microcracks, mitigating potential pathways for deleterious chlorides, but also enhances the overall structural integrity and lifespan of concrete infrastructures [61]. This chemical process often requires the presence of calcium ions (Ca^2+^), generally abundantly provided by cement-based materials, and carbonate ions (CO_3_^2−^), and depends on four main elements, namely (1) the amount of dissolved inorganic carbon, (2) system pH, (3) calcium concentration (4) and availability of nucleation sites [62,63,64]. Based on the specific conditions they are in, bacteria can bio-mineralize calcium carbonate following different paths: urealytic microorganisms use the decomposition of urea; eutrophic microorganisms consume organic matter; carbonic anhydrase-producing microorganisms accelerate the hydration of CO_2_, and nitrate-reducing microorganisms oxidize organic matter [65,66,67].

Kanwal et al. [68] developed an integrated and ecofriendly approach for corrosion inhibition of reinforced concrete by immobilizing Bacillus subtilis (BS) into carbonaceous sugarcane bagasse. The intent is to strengthen the self-healing potential of these spore-former bacteria, able to precipitate calcite, by preserving them in agricultural waste (bagasse), giving a second life to a product that has shown excellent ability in immobilizing bacteria in highly alkaline concrete environments. Tafel polarization tests proves a 97.5% efficiency of this solution, thanks to the ability of the BS to efficiently precipitate CaCO_3_, thus blocking the paths of aggressive species inside the cement paste matrix. Moreover, the immobilization of bacteria increases their survival rate with respect to their direct addiction. 

The corrosion inhibition of carbon steel rebars in concrete has been investigated also in the presence of biomolecules (BM) by bacteria cells. Indeed, Shubina et al. [69] studied the effect of 1 g·L^−1^ lipopeptide biomolecules extracted from Gram-negative bacteria cells in a simulated concrete pore solution containing chlorides. Electrochemical tests (LPR and EIS) confirm the inhibitive effects of the BM, starting from an efficiency of 58.6% that slightly increases with time. The values of R_p_ are proved higher in the presence of BM, thanks to their tendency to adhere to the carbon steel surface, by creating a physical barrier blocking the anodic and cathodic sites of the corrosion reaction. 

Erşan et al. [70,71] employed the anodic corrosion inhibitor NO_2_^−^ produced by NO_3_^−^ reducing bacteria (called “Activated Compact Denitrifying Core” ACDC), that was proven to be able to repair a 300 μm crack in 28 days. Tests were performed on rebars in cracked mortar exposed to 0.5 M Cl^−^ solution, with a mass loss equal to 50% of the one obtained on negative control specimens. Overall, microbial nitrate reduction was proven as effective as commercial chemical inhibitors like calcium nitrate.

Since the main obstacle to the MICP process in self-healing concrete is generally the concentration of soluble inorganic carbon, most studies on the topic have focused their attention on Bacillus strains, as they can form spores in the most unfriendly environments. Dissolved inorganic carbon (DIC) can influence the MICP process, since it serves as a carbon source for the bacteria and a key component in the chemical reactions that lead to the formation of calcium carbonate. It can be present in the form of bicarbonate ions and dissolved carbon dioxide within the concrete pore solution. Bacteria utilize these carbon sources as substrates for their metabolic processes. The amount of CO_3_^2−^ is dependent upon the amount of dissolved inorganic carbon and pH of the system [65,66,67]. Moreover, the presence of bicarbonate ions helps to buffer the pH of the concrete pore solution, ensuring a proper microbial activity that is pH-dependent. Moreover, these strains are Gram-positive and are characterized by *pepti-doglycan* layers that allow them to survive harsh alkaline pHs: as their spores can be dormant for decades, the problems connected to the survival of the bacterial colonies in mortar would be partially solved [72,73]. In particular, the inhibiting effects of CaCO_3_-producing bacteria were extensively investigated in the case of four *Bacillus* strains [74] tested in simulated cracked concrete with 3.5 wt.% NaCl solution. Electrochemical tests (Tafel polarization and EIS) were performed to test the inhibition efficiency of the cultures, resulting in the following order of inhibition tendency: *B. safensis* > *B. subtilis* > *B. pumilus* > *B. australimaris* > Control. The best efficiency was obtained with *B. safensis* and was 98.8% higher with respect to the control sample. This trend in corrosion inhibition arises from the ability of the cited bacteria to form a protective biofilm capable of decreasing the chloride contact with the metal and the release of metallic ions to the environment. The investigation on the effect of chlorides showed a reduction in the CaCO_3_ formation stability with increased NaCl. Nonetheless, the presence of chlorides enhanced the thickness and compactness of the biofilms.

Overall, the inhibiting effects of certain strains of bacteria on reinforced concrete exposed to chlorides have been proven to be promising, especially for the self-healing properties they impart to the material. At the same time, a more comprehensive overview of all the possible interactions between different microbial communities and concrete structures is still needed. Another source of green inhibitors are fungi. Olivia et al. [75] studied the anti-corrosive effect of fungal-based biosurfactant secreted by *Penicillium citrinum* on carbon steel bars immersed in a 0.9 wt.% NaCl solution for 100 days. The weight loss tests revealed that using 10 *v*/*v*% of biosurfactant, the lowest corrosion rate was obtained (30 μm·year^−1^), corresponding to an IE of ~58% compared to Tween 80 and the same inhibitor at different concentrations. In conclusion, the use of bacterial green inhibitors offers several noteworthy advantages. Firstly, this approach is inherently eco-friendly, aligning with green and sustainable practices by harnessing natural processes to mitigate corrosion. Moreover, live bacteria involved in the MICP process exhibit the remarkable capacity for self-healing by continuously producing calcium carbonate, thereby enabling the repair of cracks and the protection of reinforcement steel. Additionally, bacteria are a renewable resource, making their direct application in concrete corrosion inhibition a sustainable choice. Lastly, their adaptability to varying concrete conditions and responsiveness to changing environmental factors enhance their overall effectiveness.

However, it is essential to acknowledge certain challenges associated with the use of live bacteria for MICP. These include the demanding maintenance of conditions critical for bacterial survival and activity, which can be both challenging and costly. The application of live bacteria to concrete can also pose technical complexities, potentially requiring specialized equipment and expertise. Furthermore, the MICP process typically operates at a slower pace compared to chemical inhibitors, which may not provide immediate corrosion protection. Lastly, compatibility issues may arise when live bacteria interact with other chemicals used in concrete or construction applications. Nonetheless, these challenges should be weighed against the potential benefits and environmental considerations when determining the suitability of MICP for corrosion inhibition in concrete structures.

**Table 1:** List of all the green inhibitors collected in the present review. SCP stands for simulated concrete pore solution, mortar indicates rebars are embedded in mortar or concrete samples, carboxylic acid 1 refers to 3-(dimethylamino)propyl-1 ammonium stearate, carboxylic acid 2 is a fatty acid extracted from coconut and carboxylic acid 3 a fatty acid extracted from sunflower.

Substance	Test	Mix Design	IE%	[Inhibitor]	[Cl^−^]	Ref.
Rosa Damascena	SCP	/	82	12 *v*/*v*%	0.5 M NaCl	[26]
Morinda citrifolia (Noni)	Mortar	w/c = 0.45; cement/sand/gravel = 1:2:4	59	0.42 volume to cement wt.%	3 wt.% NaCl	[28]
Olive leaf extract	NaOH 0.1 M solution	/	91.9	/	0.5 M NaCl	[30]
Davidian involucrata	SCP	/	80.3	0.1 g·L^−1^	3.5 wt.% NaCl	[32]
Prosopis juliflora	SCP	/	91	100 ppm	3.5 wt.% NaCl	[33]
Fatsia japonica	SCP	/	91.2	100 mg·L^−1^	35 g·L^−1^ NaCl	[34]
Pinus resinosa extract	SCP	/	80.64	1 g·L^−1^	30 g·L^−1^ NaCl	[35]
Pinus resinosa conifer cones	SCP	/	80.64	1 g·L^−1^	30 g/L NaCl	[35]
Ginger extract	SCP	/	/	2 wt.%	0.1 M NaCl	[36]
Mango extract	SCP	/	98	2 wt.%	3.5 wt.% NaCl	[37]
Sodium phytate	SCP	/	68.3	0.01 M	0.3 M NaCl	[40]
Gossypol–indole	SCP	/	96	100 mg·L^−1^	1 M NaCl	[41]
Cactus mucilage	SCP	/	>90	0.5 *w*/*v*	16 g·L^−1^ NaCl	[42]
Bambusa Arundinacea	Mortar	w/c = 0.45; cement/sand/coarse aggregates = 1:1.2:2	/	2 cement wt.%	0.94 cement wt.% NaCl	[43]
Ricinus communis	Mortar	w/c = 0.5; cement/fine aggregates/coarse aggregates = 1:1.2:2	87	100 ppm	3.5 wt.% NaCl	[44]
Carboxylic acid 1	Mortar	w/c = 0.5; cement/sand = 1:3	96	0.1 g·L^−1^	3 cement wt.% NaCl	[45]
Carboxylic acid 2	Mortar	w/c = 0.5; cement/sand = 1:3	94	0.1 g·L^−1^	3 cement wt.% NaCl	[45]
Carboxylic acid 3	Mortar	w/c = 0.5; cement/sand = 1:3	85	0.1 g·L^−1^	3 cement wt.% NaCl	[45]
Urtica Dioica extract	SCP	/	77	0.075 wt.%	1 wt.% NaCl	[47]
Ascorbic acid	Mortar	cement/sand/water: 1/3/0.5	97.3	0.009 cement wt.%	3.29 cement wt.% NaCl	[48]
Maize gluten meal extract	Mortar	water/cement/sand: 0.6/1/3	99.72	3 cement wt.%	3 cement wt.% NaCl	[49]
Guar gum extract	Mortar	w/c = 0.45	71.1	1.4 cement wt.%	3.5 wt.% NaCl	[50]
Reed leaves	Mortar	w/c = 0.55; cement/sand = 1:3	76.98	0.5 cement wt.%	3.5 wt.% NaCl	[52]
Green tea antioxidants	Mortar	w/c = 0.54; cement/sand = 1:2.6	80	40 L·m^−3^	3.5 wt.% NaCl	[53]
Cymbopogon citratus	Mortar	w/c = 0.499; cement/sand = 1:3	99.35	0.0833 cement wt.%	3.5 wt.% NaCl	[54]
Phyllanthus muellerianus	Mortar	/	97.58	0.333 cement wt.%	3.5 wt.% NaCl	[55]
Anthocleista djalonensis	Mortar	/	97.43	0.417 cement wt.%	3.5 wt.% NaCl	[56]
Rhizophora mangle	Mortar	w/c = 0.499; cement/sand/gravel = 1:2.97:3.69	99.08	0.467 cement wt.%	3.5 wt.% NaCl	[57]
Opuntia ficus-indica	Mortar	cement/sand = 1:3	>60%	From 1.5 to 95 water v.%	3.5 wt.% NaCl	[58]
Platanus acerifolia leaves	SCP	/	99	5 wt.%	0.1 M NaCl	[59]
Platanus acerifolia leaves	SCP	/	84.25	3 wt.%	0.5 M NaCl	[60]
Bacillus subtilis in sugarcane-bagasse	Mortar	Cement/fine aggregates/coarse aggregates = 1:2.2:2.4; Water/binder = 0.4	97.5	/	5 wt.% NaCl	[68]
Lipoptide biomolecules	SCP	/	58.6	1 g·L^−1^	29.25 g·L^−1^ NaCl	[69]
NO_3_^−^ reducing bacteria	Mortar	Sand/cement/water = 3:1:0.5	/	/	0.5 M NaCl	[70]
Bacillus safensis, subtilis, pumilus, australimaris	SCP	/	98.8	/	3.5 wt.% NaCl	[74]
Penicillium citrinum antioxidant	Chloride neutral solution	/	58	10 *v*/*v*%	0.9% NaCl	[75]

## 4. Discussion and Comparison with Nitrites

For the sake of comparison, in Table 1 all the proposed green inhibitors are listed according to the main parameters of interest. Despite all these molecules being tested as corrosion inhibitors for concrete, only a few researchers have tested their efficacy directly in mortars or concrete with long exposure tests. Keeping this in mind, it is worth underlining the efficacy declared by [48] about *ascorbic acid*. In fact, only 0.009% of inhibitor vs. cement weight is sufficient to raise the efficiency (evaluated as the decrease in *i_corr_*) to 97.3% in 8-month exposure tests. This result is very interesting if compared with nitrites. The latter, in fact, can guarantee the same performances only if added in a considerably higher amount (2.6%). Another plant extract of interest is the one obtained from *Cymbopogon citratus*, a grass widely known and cultivated in Asia and India. A content of 0.083% vs. cement weight inside mortars gives an efficiency of 99.35%—beyond the inhibitive properties obtained by using 0.417% of NaNO_2_— also having beneficial effects on the mechanical properties of the tested concrete samples [54]. An interesting comparison between a green inhibitor and nitrites was offered by [46], who evaluated the effectiveness of *licorice*. Despite the lower efficiency (~30%) with respect to NaNO_2_, licorice extract was verified to be active for more than 90 days with respect to a concrete polluted by 0.8% vs. cement weight of Cl^−^. 

Eventually, the *maize gluten meal* inhibitor showed an efficiency in line with the performance of Ca(NO_2_)_2_; however, the high quantities (from 1 to 5% vs. cement weight) of the inhibitor used to preserve the state of carbon steel rebars require further tests to verify the mechanical stability of the admixture [49]. Nitrite-based corrosion inhibitors in a chloride-rich environment are the current benchmark. Their performances have been all-round tested and accepted by studies in the literature, and the cheap market price makes them a profitable option to protect carbon steel rebars embedded in reinforced concrete both from chloride attacks and carbonation-induced corrosion. They usually do not affect the mechanical properties of concrete when added in the mix design; nevertheless, depending on the specific compound employed, they can greatly impact the concrete setting time. In this regard, cement paste with a water/cement ratio of 0.3 undergo rapid setting depending on the cation employed besides nitrites. For example, calcium nitrite or magnesium nitrite accelerates up to 4%; the same occurs for potassium nitrite up to 2%, while lithium, sodium, and bismuth nitrite can reach values up to 10% [76]. This can both be an issue and an advantage depending on the specific application; nevertheless, it is a parameter to consider during the mix design. Regarding the nitrite compatibility with humans and the environment, their toxicity, which poses potential health hazards related to problems to the enzymatic systems or to some organs (kidneys and liver), has already been pointed out. On the other hand, green inhibitors should not pose by definition any kind of threat to the environment and possibly any harm to human health. The feedstock for this category of corrosion inhibitors are renewable resources, clearly offering brand new opportunities for a circular economy. Nevertheless, to extract the active ingredients with the desired functional units from the raw material, complex chemical processes can often be involved, as well as expensive or hazardous solvents (methanol, ethyl acetate, dichloromethane, hexane, and acetone). An extensive analysis on the safety of the already-mentioned green corrosion inhibitors will be of paramount importance in the future, to avoid unforeseen deleterious effects for the human safety. The effects on the mechanical properties of only a few green corrosion inhibitors has been evaluated up to this date, providing satisfactory results comparable with nitrite compounds [28,44,46,51]. However, the vast majority of the green inhibitors mentioned in this review still need further studies on the topic, and this will play a key role in the selection process of a valid alternative to nitrites. Indeed, organic compounds usually tend to be detrimental for the mechanical properties of concrete when added in the mix design phase. This could lead, in some cases, to a trade-off between mechanical and corrosion performances when green inhibitors are involved. 

Concerning corrosion-inhibitive performances, the declared efficiency of many green inhibitors is on par with nitrite compounds, at least in the short term. However, the efficiency is often evaluated through electrochemical measures carried out in solution rather than for rebars embedded in mortars, leading to possible substantial deviation from real conditions.

## 5. Conclusions

The need to find effective substances for the control of rebars corrosion in concrete with a low level of toxicity, economic affordability and an overall low environmental impact allows many researchers to focus their efforts on the testing of many green molecules both of vegetal and non-vegetal origin. Based on the present survey, the following conclusions can be drawn:

- Very recently, many organic molecules have been extracted from plants and leaves. Thanks to the presence of heteroatoms (O, S and N), aromatic rings and other polar groups, the majority of those substances have been found to donate electron density to the empty *d*-orbital of Fe, sometimes also accumulating in correspondence of defects or pits. Generally, all the molecules tested demonstrate mixed or at least anodic-inhibitive properties.
- Very few recent studies have compared the effectiveness of green inhibitors in a concrete pore solution (SCP) with carbon steel embodied in concrete or mortar. As a result, despite the declared inhibitive efficiency (IE%) seeming to well compete with nitrites, long term tests in cementitious materials should be performed for a reliable comparison.
- Various waste materials could be efficiently employed as stockpile to produce low-carbon-footprint mixed-type corrosion inhibitors for concrete. However, this kind of study still needs further development to evaluate, in more detail, the real effectiveness of these green inhibitors, which often display an underwhelming efficiency in terms of corrosion rate reduction.
- Based on the proposed inhibition efficiencies and completeness of the studies among all the chemicals collected, the following are considered as promising alternatives to nitrites: *ascorbic acid*, *Cymbopogon citratus extract*, *licorice extract* and *maize gluten meal*.
- Despite several solutions presenting inhibitory properties comparable to the ones of commercially available products like nitrites, often the high quantity of cement weight used poses a concern to the structural integrity of the mix, requiring mechanical tests to be performed. Moreover, some of the tested molecules, despite the environmental compatibility, do not guarantee an adequate level of protection, as the resulting corrosion rates are far from the levels compatible with the design life of common structures.

## Data Availability

Data will be provided upon reasonable request to the corresponding author.
